# A multicenter phase II trial of paclitaxel, carboplatin, and cetuximab followed by chemoradiotherapy in patients with unresectable locally advanced squamous cell carcinoma of the head and neck

**DOI:** 10.1002/cam4.2852

**Published:** 2020-01-13

**Authors:** Tomohiro Enokida, Takenori Ogawa, Akihiro Homma, Kenji Okami, Shujiro Minami, Ayako Nakanome, Yasushi Shimizu, Daisuke Maki, Yuri Ueda, Takao Fujisawa, Atsushi Motegi, Akira Ohkoshi, Jun Taguchi, Koji Ebisumoto, Shogo Nomura, Susumu Okano, Makoto Tahara

**Affiliations:** ^1^ Department of Head and Neck Medical Oncology National Cancer Center Hospital East Chiba Japan; ^2^ Department of Otolaryngology – Head and Neck Surgery Tohoku University School of Medicine Miyagi Japan; ^3^ Department of Otolaryngology, Head & Neck Surgery Hokkaido University Graduate School of Medicine Sapporo Japan; ^4^ Department of Otolaryngology – Head Neck Surgery Tokai University Kanagawa Japan; ^5^ Department of Otorhinolaryngology National Hospital Organization Tokyo Medical Center Tokyo Japan; ^6^ Department of Medical Oncology Hokkaido University Hospital Sapporo Japan; ^7^ Division of Radiation Oncology and Particle Therapy National Cancer Center Hospital East Chiba Japan; ^8^ Biostatistics Division Center for Research Administration and Support National Cancer Center Chiba Japan

**Keywords:** carboplatin, cetuximab, chemoradiotherapy, induction chemotherapy, paclitaxel, unresectable locally advanced squamous cell carcinoma of the head and neck

## Abstract

**Background:**

Induction chemotherapy (IC) in locally advanced squamous cell carcinoma of the head and neck (LA‐SCCHN) often compromises compliance with subsequent chemoradiotherapy (CRT), which negatively affects outcomes. Here, we assessed the combination of paclitaxel (PTX), carboplatin (CBDCA), and cetuximab (Cmab) as IC for unresectable LA‐SCCHN.

**Methods:**

Induction chemotherapy consisted of weekly CBDCA area under the plasma concentration‐time curve = 1.5, PTX 80 mg/m^2^ and Cmab with an initial dose of 400 mg/m^2^ followed by 250 mg/m^2^ for 8 weeks. Following IC, CDDP (20 mg/m^2^, 4 days × 3 cycles) and concurrent radiotherapy (70 Gy/35 fr) were started. Primary endpoint was the proportion of CRT completion (%CRT completion). PCE was planned to be deemed effective if the Bayesian posterior probability (PP), defined as the probability that %CRT completion was larger than the threshold value of 65%, exceeded 84%.

**Results:**

Thirty‐five patients were enrolled. Cases were hypopharynx/oropharynx/larynx in 17/17/1 patients, all at Stage IV. Of 35 patients, 34 (97%) completed IC and 32 received CRT and met the criteria of full analysis set (FAS). In FAS, the %CRT completion was 96.9%, and PP was 99.9%, exceeding the prespecified boundary of 84%. Mean cumulative dose and relative to dose intensity of CDDP in CRT was 232.5 mg/m^2^ and 100%, respectively. Response rate was 88.6% by IC and 93.8% in the CRT phase. Three year overall survival was 83.5%. Main grade 3 toxicities included neutropenia (11.4%) and skin rash (5.7%) during IC; and oral mucositis (31.3%) and neutropenia (12.5%) during CRT. No grade 4 toxicity or treatment‐related death was seen.

**Conclusions:**

PCE as IC was feasible, with promising efficacy and no effect on compliance with subsequent CRT in unresectable LA‐SCCHN.

## Graphical abstract

The combination of PCE as induction chemotherapy was feasible and had no effect on compliance with subsequent chemoradiotherapy (CRT) with CDDP, and provided encouraging efficacy in high‐risk unresectable locally advanced squamous cell carcinoma of the head and neck (LA‐SCCHN). The regimen is a favorable alternative to TPF for LA‐SCCHN patients who required more aggressive treatment, herein sequential CRT.

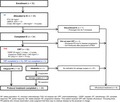

## 1 BACKGROUND

Head and neck cancers (HNC) are the sixth‐most common cancer in the world, and approximately 650 000 new cases are projected annually.^1^ An estimated 60% of these patients present with locally advanced disease (stage III/IV). Standard treatment for unresectable locally advanced squamous cell carcinoma of the head and neck (LA‐SCCHN) is concurrent chemoradiotherapy (CRT).^2^ However, a significant number of cases will recur, particularly those with higher nodal status at presentation, indicating a clear need for further therapeutic intervention in this population.^2^, ^3^

Induction chemotherapy (IC) may improve the prognosis of LA‐SCCHN.^4^, ^5^ Several studies have shown that IC consistently results in higher response and exerts a pronounced effect on distant metastases.^6^, ^7^ In several phase III trials, combination of docetaxel, cisplatin (CDDP), and 5‐fluorouracil (TPF) improved clinical response and survival compared with CDDP and 5‐fluorouracil (PF) alone, and this regimen is now considered the accepted standard of care for IC.^8^, ^9^, ^10^, ^11^ However, because no study featured CRT with CDDP in the control arm, the addition of IC (TPF) to CDDP‐based CCRT (sequential CRT) has not been shown to be superior to CDDP‐based CCRT alone.^8^, ^9^, ^12^, ^13^, ^14^, ^15^, ^16^ More importantly, discussion continues over whether sequential CRT can be safely administered and whether its compliance can be assured given the significant toxicities of induction TPF. In TAX 324, 21% of patients (49/255 patients) did not proceed to per‐protocol carboplatin (CBDCA) plus RT,^17^ while in TAX323, approximately 25% of patients did not complete all cycles of full‐dose TPF. Additionally, induction TPF‐associated death is reported up to 5%.^18^ Since CRT is the definitive standard treatment, an obvious concern is that aggressive treatment, herein induction TPF followed by CRT with CDDP, might not ultimately improve outcomes if the entire treatment, especially the CRT component, cannot be completed.

The combination of paclitaxel (PTX), CBDCA, and cetuximab (Cmab) (PCE) as IC has been tested in phase II trials and used in daily practice with excellent efficacy (overall response rates [ORRs] to the IC ranging between 65% and 97%) and manageable toxicity safety (no deaths occurred during IC).^19^, ^20^, ^21^ Nevertheless, prospective data on PCE as IC followed by CRT with CDDP as per study design in a heterogeneous population of patients with unresectable LA‐SCCHN have yet to appear.

Accordingly, we conducted a phase II study to assess the feasibility—with a primary focus on compliance with CRT—and efficacy of induction PCE for those with highly aggressive disease, which we often experience in daily practice.

## 2 PATIENTS AND METHODS

See Supporting Information for more details.

### 2.1 Patients

For inclusion, patients were required to meet all of the following criteria: histologically proven squamous cell carcinoma; primary lesion located at the oropharynx, hypopharynx or larynx; and unresectable locally advanced HNC that fulfills at least one of the following conditions: (a) primary lesion or cervical lymph node metastasis invasion to the carotid artery, cranial base, or cervical vertebrae; (b) cervical lymph node metastasis of N2b involving the lower neck (Level IV or supraclavicular lymph node), N2c or N3 (UICC⁄TNM, 7th edition); or (c) T4 primary lesion located at the oropharynx.

### 2.2 Treatment and assessment

The protocol treatment consisted of IC followed by concurrent CRT, and salvage surgery if applicable. First, patients received IC consisting of CBDCA area under the plasma concentration‐time curve (AUC) = 1.5, PTX 80 mg/m^2^ and Cmab with an initial dose of 400 mg/m^2^ followed by 250 mg/m^2^ administered weekly for 8 weeks. If the physician omitted a cytotoxic drug (CBDCA or PTX), they could continue PCE until the number of administrations of cytotoxic drug (either CBDCA or PTX) reached eight, within 10 weeks after the start of IC. Prophylactic use of granulocyte‐colony stimulating factor (G‐CSF) was permitted if PCE was not given due to neutropenia in the preceding course. Following IC, CDDP and concurrent radiotherapy were started. Chemotherapy consisted of a 2‐hour infusion of CDDP at a dose of 20 mg/m^2^/d on days 1‐4, repeated three times at 3‐week intervals, giving a planned total CDDP dose during CRT of 240 mg/m^2^. Radiation therapy was carried out once daily with 70 Gy/35 fractions over 7 weeks using high‐energy photons of 4‐10 MV X‐rays and intensity‐modulated radiotherapy planning, starting on day 1. Objective response was evaluated using the modified RECIST criteria.

### 2.3 Study design

The study was conducted under a multicenter, prospective, single‐arm phase II design to assess the feasibility and efficacy of PCE as IC for unresectable LA‐SCCHN. Our primary purpose was to assess whether induction PCE compromises compliance with subsequent CDDP‐based CRT. The study protocol was approved by the institutional review board of each participating institution and registered with the UMIN Clinical Trials Registry, number UMIN000014430. Primary endpoint was the proportion of CRT completion (%CRT completion), defined by (a) completion of planned CDDP relative to dose intensity (RDI) ≥80%; and (b) completion of radiotherapy within 2 weeks after the planned completion date.

### 2.4 Statistical analysis

Our primary aim was to examine whether compliance with CDDP‐based CRT is not worse when experimental PCE is given as IC. During planning, we retrospectively collected individual data of 75 patients who had undertaken CDDP‐based CRT without any IC at an institution of the first author (National Cancer Center Hospital East), and found that %CRT completion was 81% (see Doc. S1 for background information on the cohort). Accordingly, the threshold and expected values of %CRT completion were set as 65% and 80%, respectively. When Bayesian posterior probability (PP) that %CRT completion exceeds 65% was more than 84%, PCE as IC was planned to be deemed effective, or otherwise ineffective. Using the weakly informative beta distribution of *Beta*(1,1) as prior distribution, required sample size of the full analysis set (FAS) was calculated as 31.^22^ Numerical simulation with 10 000 iterations showed type‐I and type‐II error rates of 18.9% and 15.3%, respectively. Full analysis set was defined to include patients who accomplished induction PCE therapy within the protocol‐defined dose reduction criteria and who were treated with CDDP‐based CRT at least once. Considering that a few patients might not meet FAS criteria, we enrolled 4‐5 additional patients. When the number of patients meeting the FAS criteria exceeded 31, the same decision criteria for declaring efficacy was planned to be used.

## 3 RESULTS

### 3.1 Patients and disease characteristics

From July 2014 to July 2017, 35 eligible patients were accrued from 5 sites (32 males and 3 females; median age 63 years). Characteristics and stage distribution are listed in Table 1 and Figure S1, respectively. All patients had neck lymph node involvement with low neck N2b, and N2c or worse. The most common primary site was the oropharynx and larynx (both 49%, 17/35). p16 staining as a surrogate for human papilloma virus (HPV) was reported as an addendum to pathology reports from patient specimens. Nine (53%) of 17 patients tested positive. A total of 32 patients (91.4%) were current drinkers and 30 (85.7%) had a history of tobacco use, of whom 90% had a ≥10 pack‐year smoking history. Accordingly, at least 88% of oropharyngeal cancer patients among FAS cases (14/16 patients) were considered either intermediate‐ or high‐risk populations, as defined by the Radiation Therapy Oncology Group (RTOG) 0129 criterion^23^ (Figure S2). All patients underwent prophylactic percutaneous endoscopic gastrostomy feeding tube placement before starting CRT.

**Table 1:** Patient demographics and clinical characteristics

Characteristic	SP^a^ (n = 35)		FAS (n = 32)	
	No. of patients	%	No. of patients	%
Median age (range)	63 (41‐72)	—	63.5 (41‐72)	
Sex				
Male/female	32/3	91.4/8.6	29/3	90.6/9.4
Staging				
Stage IVa/IVb	27/8	77.1/22.9	24/8	75/25
T4	18	51.4	17	53.1
N3	4	11.4	4	12.5
Site of primary tumor				
Oropharynx	17	48.6	16	50.0
Hypopharynx	17	48.6	15	46.9
Larynx	1	2.9	1	3.1
p16 status for oropharyngeal cancer				
p16‐positive	9	25.7	9	28.1
p16‐negative	2	5.7	2	6.3
Unknown	6	17.1	5	15.6
Reason for unresectability				
Inoperable	10	28.6	10	31.3
N status	25	71.4	23	71.9
T4 oropharyngeal origin	11	31.4	10	31.3
RTOG 0129 risk group for oropharyngeal cancer^23^				
Low risk	0	0	0	0
Intermediate or high risk^b^	15	88.2	14	87.5
Low risk or high risk^b^	2	11.8	2	12.5
Smoking status				
Never	5	14.3	5	15.6
Former	15	42.9	15	46.8
Current	15	42.9	12	37.5
Cigarette smoker^c^ (pack years)				
<10	3	10	3	11.5
≥10	27	90	23	88.5
Smoking consumption [pack years] mean ± SD (range)	24.3 ± 20.3 (0‐76.5)	—	23.9 ± 21.1 (0‐76.5)	—
Alcohol status				
Never	3	8.6	4	12.5
Former	0	0	0	0
Current	32	91.4	28	87.5
Alcohol consumption^d^ [drink/wk] mean ± SD (range)	14.7 ± 16.2 (0‐56)	—	15.0 ± 16.9 (0‐56)	—

Abbreviations: FAS, full analysis set; SD, standard deviation.

a Safety population (equivalent to total population).

b Depending on p16 status.

c Among former or current smokers.

d Data were available for 33 of 35 patients. One drink contains 10 g of pure alcohol.

#### 3.1.1 Treatment and CRT completion rate

**Figure 1:**
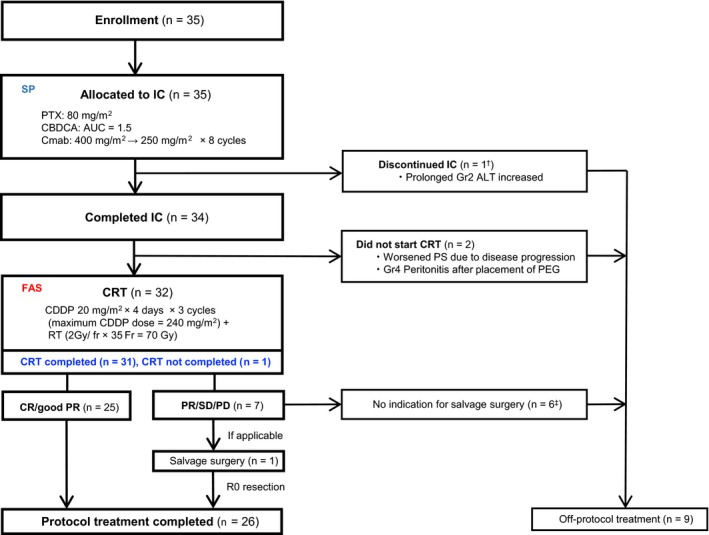
Patient flow diagram of treatment delivery. CDDP, cisplatin; CRT, chemoradiotherapy; CR, complete response; FAS, full analysis set; IC, induction chemotherapy; PD, progressive disease; PR, partial response; RT, radiotherapy; SD, stable disease; SP, safety population. ^†^This patient received CRT with CDDP off protocol. ^‡^Including three PR patients who choose observation under judgment that there was no residual disease by the physician in charge

All 35 enrolled patients proceeded to IC (safety population; SP) (Figure 1). Of 35 SP patients, 34 completed IC. One discontinued IC due to prolonged grade 2 serum alanine aminotransferase (ALT) elevation. A majority of the patients received the full course of induction PCE as planned per protocol in terms of the number of drug administrations and dose intensity (Table S1). Three patients (8.6%) received G‐CSF support after omission of treatment due to neutropenia in a prior cycle. Two of 34 patients who completed IC did not start CRT, one each due to disturbed performance status caused by disease progression and peritonitis related to placement of the PEG. In total, 32 received CRT (FAS).

In FAS, mean cumulative dose and RDI of CDDP during CRT was 232.5 mg/m^2^ (range: 160‐240 mg/m^2^) and 100% (range: 66.7%‐100%), respectively (Table 2). Only one patient did not receive CDDP above 200 mg/m^2^ due to grade 3 mucositis and impaired PS from 0 to 2. The remaining patient omitted radiotherapy on 1 day due to mucosal infection. All FAS cases completed planned radiotherapy. Thus, %CRT completion was 96.9% (31/32, 95% CI, 83.8%‐99.9%, 95% credible interval, 86.3%‐99.8%). Bayesian PP was 99.9%, which exceeded the prespecified cutoff value of 84%, and the primary objective of this study was therefore met (Table 2).

**Table 2:** Compliance with chemoradiotherapy

	No. of patients (n = 32^a^)
Cisplatin (CDDP)	
No. of patients with ≥RDI 80	31
CDDP RDI median (range)	100 (66.7‐100)
Cumulative CDDP dose [mg/m^2^] mean ± SD (range)	232.5 ± 17.2 (160‐240)
Radiotherapy (RT)	
RT dose [Gy] mean ± SD	70 ± 0.0
Omission of RT	
No (%)	31 (96.9%)
Yes (%)	1 (3.1%)
Total days of omission of RT (d)	1
Rate of treatment completion (%) (95% CI)	96.9 (83.8‐99.9)
Posterior probability (PP)	99.9% (>84%)

Abbreviations: CI, confidence interval; RDI, relative dose intensity; SD, standard deviation.

a FAS (full analysis set).

### 3.2 Treatment outcomes

**Figure 2:**
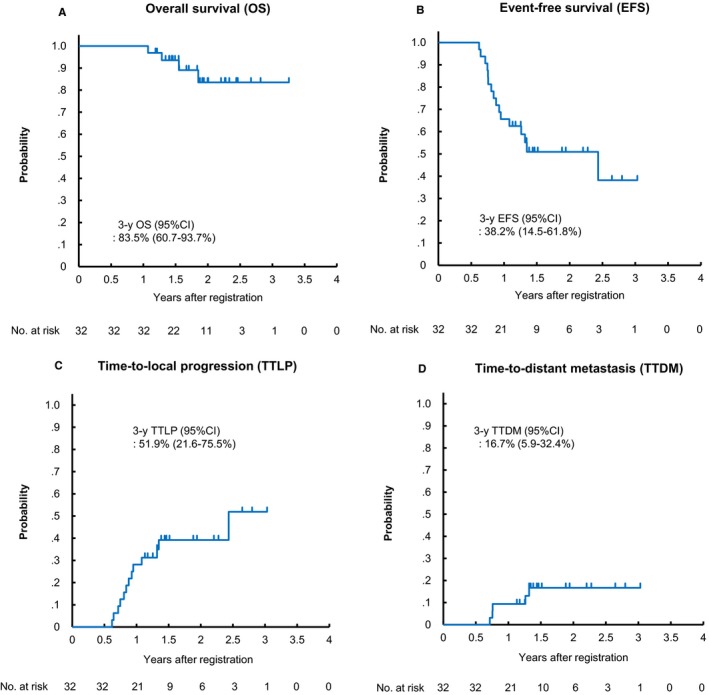
Patient (A) overall survival, (B) event‐free survival, (C) time‐to‐local progression, and (D) time‐to‐distant metastasis of patients treated with the IC‐PCE followed by CRT (FAS). CI, confidence interval. CRT, chemoradiotherapy; FAS, full analysis set; IC, induction chemotherapy; PCE, paclitaxel, carboplatin, and cetuximab

**Figure 3:**
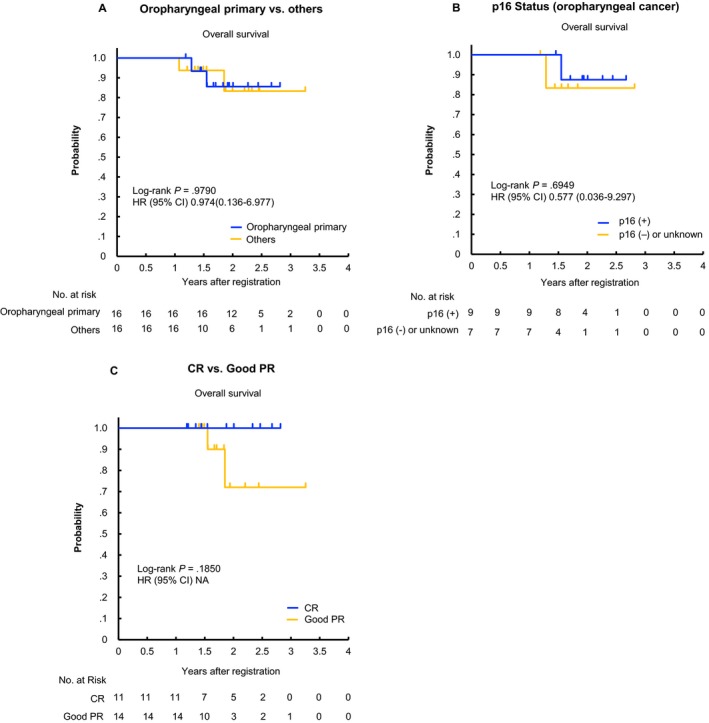
Overall survival stratified according to (A) primary site, (B) p16 status among oropharyngeal cancers and (C) CR vs good PR in the FAS population. CI, confidence interval; CR, complete response; FAS, full analysis set; HR, hazard ratio; PR, partial response; NA, not available

Efficacy data are listed in Table 3. All enrolled patients were assessable for response at least once. Overall response rate was 88.6% (31/35), with 0 CR and 31 PR in the IC phase; and 93.8% (31/32), with 11 CR, 14 good PR, and 5 PR in the CRT phase, respectively. Accordingly, clinical complete remission rate at CRT completion was 78.1% (25/32). After a median follow‐up of 1.89 years (range: 1.19‐3.26 years) for FAS, 3‐year OS was 83.5% with a 3‐year event‐free survival (EFS) of 38.2%, 3‐year time‐to‐local progression (TTLP) of 51.9% and 3‐year time‐to‐distant metastasis (TTDM) of 16.7% (Figure 2; prognosis data of SP are presented in Figure S3). In survival analyses according to oropharyngeal primary vs others, p16 status among oropharyngeal cancer patients and CR vs good PR showed no statistically significant difference in clinical outcomes (Figure 3). At data cut‐off, 16 patients had disease progression, including locoregional site disease only (n = 11), distant metastatic disease (n = 3), or both (n = 2) (Figure S4). Among them, one patient who did not achieve CR or good PR at the time of CRT completion received R0 salvage surgery as protocol treatment, and the other five received off‐protocol salvage surgery for late locoregional recurrence. Accordingly, six (37.5%) of the 16 patients received salvage surgery with curative intent because of the absence of distant metastasis (DM): five received salvage neck dissection for cervical node disease and one received salvage laryngectomy for local recurrence of hypopharyngeal cancer. Systemic chemotherapy was carried out in nine patients as first‐line treatment for disease progression, all of which were off‐protocol.

**Table 3:** Response to treatment

CR	Good PR	PR	SD	PD	%CR (95% CI)	%RR (95% CI)
*Induction chemotherapy (n = 35)*						
0	—	31	3	1	0 (NA)	88.6 (73.3‐96.8)
*Chemoradiotherapy (n = 32)*						
11	14	5	1	1	78.1 (60.0‐90.7)	93.8 (79.2‐99.2)

Note

%RR, proportion of CR+PR; %CR, proportion of CR+good PR.

Abbreviations: CI, confidence interval; CR, complete response; NA, not available; PD, progressive disease; PR, partial response; SD, stable disease.

### 3.3 Toxicity

Overall toxicities during IC and CRT are listed in Tables 4 and 5, respectively. The most common grade 3 toxicity during IC was neutropenia (11.4%), followed by leukopenia (8.6%), rash (5.7%), and anemia (5.7%). During CRT, the most common grade 3 toxicities were mucositis (31.3%), radiation dermatitis (12.5%), neutropenia (12.5%), leukopenia (12.5%), and dysphagia (9.4%). There were no instances of febrile neutropenia (FN), and no grade 4 toxicity or treatment‐related death. Total frequency of grade 3 or more toxicity was 14.2% in IC and 43.6% in CRT. Late toxicity was evaluable in 29 cases, and median time from completion of treatment to evaluation was 12 months (range: 6‐29). The most common late toxicities were dry mouth (72.4%) and dysgeusia (51.7%), but most of these were grade 1 or 2. Total frequency of grade 3 or more late toxicity was 10.3% (Table S2).

**Table 4:** Selected toxicity during induction chemotherapy

	n = 35^a^		
	All grade	Grade3	Grade4
Hematologic			
Leukopenia (%)	32 (91.4)	3 (8.6)	0 (0)
Neutropenia (%)	28 (80)	4 (11.4)	0 (0)
Anemia (%)	24 (68.6)	2 (5.7)	0 (0)
Thrombocytopenia (%)	1 (2.9)	0 (0)	0 (0)
Febrile neutropenia (%)	0 (0)	0 (0)	0 (0)
Non‐hematologic			
Infusion reaction (%)	3 (8.6)	1 (2.9)	0 (0)
Allergic reaction (%)	3 (8.6)	1 (2.9)	0 (0)
Nausea (%)	3 (8.6)	0 (0)	—
Anorexia (%)	6 (17.1)	1 (2.9)	0 (0)
Mucositis (%)	11 (31.4)	0 (0)	0 (0)
Fatigue (%)	19 (54.3)	1 (2.9)	—
Constipation (%)	13 (37.1)	0 (0)	0 (0)
Peripheral neuropathy (%)	14 (40.0)	1 (2.9)	0 (0)
Alopecia (%)	27 (77.1)	—	—
Rash (%)	32 (91.4)	2 (5.7)	0 (0)
Paronychia (%)	12 (34.3)	0 (0)	—
Other skin^b^ (%)	23 (65.7)	0 (0)	0 (0)
Pneumonitis (%)	1 (2.9)	0 (0)	0 (0)
Pneumonia (%)	1 (2.9)	1 (2.9)	0 (0)
Soft tissue infection (%)	2 (5.7)	0 (0)	0 (0)
Hypomagnesemia (%)	12 (34.3)	0 (0)	0 (0)
Acute kidney injury (%)	1 (2.9)	0 (0)	0 (0)
AST elevation (%)	8 (22.9)	0 (0)	0 (0)
ALT elevation (%)	21 (60.0)	0 (0)	0 (0)
Total with ≥Grade 3 toxicity	5 (14.2)		

Note

Graded according to common toxicity criteria for adverse events version 4.0.

Abbreviations: ALT, alanine aminotransferase; AST, aspartate amino transferase.

a SP (safety population).

b Including seborrheic dermatitis, dry skin, pruritus and skin cracks.

**Table 5:** Selected toxicity during chemoradiotherapy

	n = 32^a^		
	All grades	Grade 3	Grade 4
Hematologic			
Leukopenia (%)	32 (100)	4 (12.5)	0 (0)
Neutropenia (%)	28 (87.5)	4 (12.5)	0 (0)
Anemia (%)	31 (96.9)	2 (6.3)	0 (0)
Thrombocytopenia (%)	11 (34.4)	0 (0)	0 (0)
Febrile neutropenia (%)	0 (0)	0 (0)	0 (0)
Non‐hematologic			
Radiation dermatitis (%)	30 (93.8)	4 (12.5)	0 (0)
Mucositis (%)	32 (100)	10 (31.3)	0 (0)
Dysgeusia (%)	31 (96.9)	—	—
Dysphagia (%)	18 (56.3)	3 (9.4)	0 (0)
Dry mouth (%)	26 (81.3)	0 (0)	—
Mucosal infection (%)	2 (6.3)	0 (0)	0 (0)
Soft tissue infection (%)	2 (6.3)	1 (3.1)	0 (0)
Pneumonia (%)	1 (3.1)	0 (0)	0 (0)
Nausea (%)	16 (50)	1 (3.1)	—
Anorexia (%)	14 (43.8)	1 (3.1)	0 (0)
Fatigue (%)	14 (43.8)	0 (0)	—
Peripheral neuropathy (%)	13 (40.6)	0 (0)	0 (0)
Acute kidney injury (%)	11 (34.4)	0 (0)	0 (0)
AST elevation (%)	7 (21.9)	0 (0)	0 (0)
ALT elevation (%)	15 (46.9)	0 (0)	0 (0)
Hypomagnesemia (%)	14 (43.8)	0 (0)	0 (0)
Total with ≥Grade 3 toxicity	14 (43.6)		

Note

Graded according to common toxicity criteria for adverse events version 4.0.

Abbreviations: ALT, alanine aminotransferase; AST, aspartate amino transferase.

a FAS (full analysis set).

## 4 DISCUSSION

This phase II trial evaluated induction PCE and concurrent CRT with CDDP as per study design in a heterogeneous group of patients with unresectable LA‐SCCHN. Results showed the high feasibility of subsequent therapy, represented by a %CRT completion of 96.9%. In addition, considerable efficacy was seen, with a %CR at CRT completion of 78.1% and 3‐year OS of 83.5% in a patient population harboring far‐advanced disease with a heavy smoking and drinking history.

The question of whether TPF followed by concurrent CRT with CDDP can be safely administered with assured compliance has been discussed. The prospective phase II, single‐arm Southwest Oncology Group study (S0216) treated 74 LA‐SCCHN patients with two cycles of induction TPF, followed by concurrent CRT with CDDP 100 mg/m^2^ on days 1 and 22.^24^ Despite two cycles of CDDP during CRT, only 50 (68%) of 74 patients completed all planned treatment; notably, 11 (18%) of 61 patients who started CRT could not finish. In our study, in contrast, 34 of 35 patients (97.1%) completed induction PCE, and only one of 32 FAS patients (3%) who started CRT were unable to complete it owing to toxicity. Moreover 63 patients (85%) in S0216 experienced grade 3 or higher toxicities, including 13 (18%) who required hospitalization for FN under prophylactic ciprofloxacin and two treatment‐related deaths, one each due to FN and a cardiac cause during IC. Furthermore, there were two additional toxicity‐related deaths during CRT, one from FN and the second from a cardiac cause. In contrast, we saw no FN, grade 4 toxicity or treatment‐related death in the present study, even though primary prophylactic G‐CSF support and prophylactic antibiotics were not permitted, and use of secondary prophylactic G‐CSF against neutropenia (8.3%) was limited throughout the IC phase. Grade 3 or higher toxicity was much less frequent than in S0216 (14.2% vs 85% in IC, 43.6% vs 91% in CRT, respectively). These findings are more frequently seen in daily practice. Guguillaume et al retrospectively reported toxicities during three or four cycles of induction TPF in LA‐SCCHN^25^: among patients with induction TPF, 11% discontinued IC at the first cycle of TPF and 15% discontinued IC at the second cycle of TPF, primarily due to treatment‐related toxicity. Moreover 36.1% experienced renal failure (grade 3 in 14.7%), which often and directly compromised subsequent CRT (vs grade 1 in 2.9% in the current study). In addition, 37% developed diarrhea, likely due to continuous infusion of fluorouracil, whereas no patient experienced diarrhea in our present study. The excellent toxicity profile of induction PCE would likely lead to excellent compliance of subsequent CRT with CDDP. Although IC was added prior to CRT, 96.9% achieved more than 200 mg/m^2^ CDDP during CRT, which was considered an appropriate cumulative dose to show a significant survival benefit compared with RT alone independent of CDDP schedule.^26^, ^27^ Very recently, Haddad et al reported a phase II clinical trial with randomization to PCE and combination of Cmab and TPF (C‐TPF), followed by treatment at the local physician's discretion in patients with LA‐HNSCC.^28^ Mean RDI during PCE in their study was similar to that in our present study (Table S1), with manageable toxicities and no treatment‐related deaths. Moreover the number of patients who received CRT with CDDP as post‐IC local therapy (n = 15, not as per study design) was significantly higher in the PCE versus C‐TPF arm (52% vs 20%; *P* = .001), suggesting increased toxicity with CDDP and RT in the post‐TPF setting. Additionally, unexpected RT omission and accompanying prolongation of radiation treatment time, which negatively affect local control and survival in patients treated with CRT,^29^ were observed in only one patient and by one day. As CRT is the standard therapy for unresectable LA‐SCCHN and increases cure rates compared with RT alone, preservation of the intended treatment plan and the ability to receive sufficient CDDP and a full dose of RT should help maximize favorable outcomes. Given this, the current PCE regimen followed by CRT with CDDP is a well‐balanced treatment.

Although induction PCE was less toxic than TPF and the patients had far advanced disease, response to IC (ORR 88.6%) was preserved; RR to TPF in TAX 323 and TAX 324 was 68% and 72%, respectively. Furthermore, the 3‐year OS of 83.5% is a promising result for an unresectable LA‐SCCHN population; 2‐year OS in the TPF arm in TAX 323 and 3‐year OS to the CRT with CDDP (without prior IC) arm in the Adelstein study was 43% and 37%, respectively.^2^, ^9^ The appearance of DM after definitive CRT in LA‐SCCHN is almost invariably fatal: the overall 5‐year survival rate for patients who developed DM after CRT is 0% vs 51.6% for patients without DM.^30^ Induction chemotherapy in patients with a high risk of DM appear to gain certain benefits from the sequential CRT approach, since IC offers theoretical benefits with the potential to reduce the risk of distant metastases by eradication of micrometastatic disease, which can confer a survival benefit. This effect was particularly expected in patients with advanced nodal disease, such as multiple involved large‐volume nodal disease, and low nodes.^31^, ^32^ Bhattasali et al reported the significance of nodal status from the viewpoint of IC in p16‐positive oropharyngeal cancer.^33^ Despite a favorable effect of p16‐positivity, patients with low neck N2b/N2c, or N3 cervical lymphadenopathy who received CRT alone experienced a higher rate of DM and a trend toward worse survival compared with docetaxel and platinum‐based IC (TPF or PF) followed by CRT: 3‐year DM was 38% vs 18% (adjusted hazard ratio [HR] = 0.32 [95%CI, 0.13‐0.82]), and 3‐year overall survival was 67% vs 83% (adjusted HR = 0.48 [95%CI, 0.21‐1.12]). Although an unadjusted indirect comparison, these data may suggest that our induction PCE has similar efficacy to conventional docetaxel and platinum‐based IC on reducing DM, and demonstrate improved survival in this patient population, who are at high risk of DF: 3‐year TTDM and OS in our present study was 16.7% and 83.5%, respectively. Additionally, half of our FAS cases had a hypopharyngeal primary, which is considered a high‐risk factor for DM after CRT,^30^ which therefore also supports this assumption.

In contrast to tobacco‐related squamous cancers, the prognosis of HPV‐related cancers is favorable irrespective of the fundamental treatment approach.^23^, ^34^, ^35^, ^36^ According to the RTOG 0129 criterion, which is based on the TNM classification and smoking status of LA‐SCCHN patients treated with CRT with high‐dose CDDP (100 mg/m^2^ on days 1, 22, and 43) alone,^23^ at least 88% (14/16) of oropharyngeal cancers of the FAS cases were considered to represent either an intermediate or high‐risk population due to their heavy smoking and advanced nodal status. Given that their 3‐year OS was expected to range from 46.2% to 70.3% when treated with CRT alone, the addition of induction PCE prior to CRT with CDDP might improve prognosis in these high‐risk oropharyngeal cancer patients (2‐year OS in the oropharyngeal cancer group: 85.6% in Figure 3A). Additionally, many patients in our study were active drinkers (87.5%, 28/32 of FAS cases), which is also associated with poor survival among SCCHN patients treated CRT.^37^ This also indicates that our enrolled patients were definitely at high risk for cancer death when treated with CRT alone, and accordingly represent a patient population who need additional treatment, herein IC.

We evaluated the significance of “good PR” to avoid unnecessary additional therapy after the completion of CRT. In a phase II study of the efficacy and safety of CRT with CDDP plus S‐1 in patients with unresectable LA‐SCCHN, patients who achieved CR or good PR had significantly better survival than those who did not.^38^ Although our present study saw no statistically significant difference between CR and good PR in terms of survival, this was a subgroup analysis in a small number of enrolled patients. It should therefore be evaluated with particular care, and warrants further investigation.

## 5 CONCLUSION

In this phase II trial, we found that PCE as IC was feasible and had no effect on compliance of subsequent CRT with CDDP. This in turn suggests that this well‐balanced strategy provides considerable efficacy and encouraging survival in a patient population with far advanced and highly aggressive disease, including high‐risk oropharyngeal cancer. We consider that these results indicate that induction PCE is a favorable alternative to induction TPF in daily clinical practice for patients with unresectable LA‐SCCHN who require more aggressive treatment, herein sequential CRT.

## CONFLICT OF INTEREST

KO, SO, and MT received honoraria from Merck Serono. The remaining co‐authors declare that the research was conducted in the absence of any commercial or financial relationships that could be construed as a potential conflict of interest.

## AUTHOR CONTRIBUTIONS

TE contributed to conceptualization, project administration, and writing‐review and editing. TO, AH, KO, SM, AN, YS, DM YU, TF, AM, and SO contributed to project administration and resources. SN contributed to statistical analysis and writing‐original draft and editing. MT contributed to conceptualization, funding acquisition, project administration, resources, and supervision.

## Data Availability

The data that support the findings of this study are available from the corresponding author upon reasonable request.
