# Supplementary material for: A multicenter phase II trial of paclitaxel, carboplatin, and cetuximab followed by chemoradiotherapy in patients with unresectable locally advanced squamous cell carcinoma of the head and neck
Source: Cancer Med. 2020 Jan 13;9(5):1671–82. doi: 10.1002/cam4.2852 (PMC7050099; doi:10.1002/cam4.2852)
Supplement: Supplementary file 1 [file CAM4-9-1671-s001.pptx]

## Slide 1
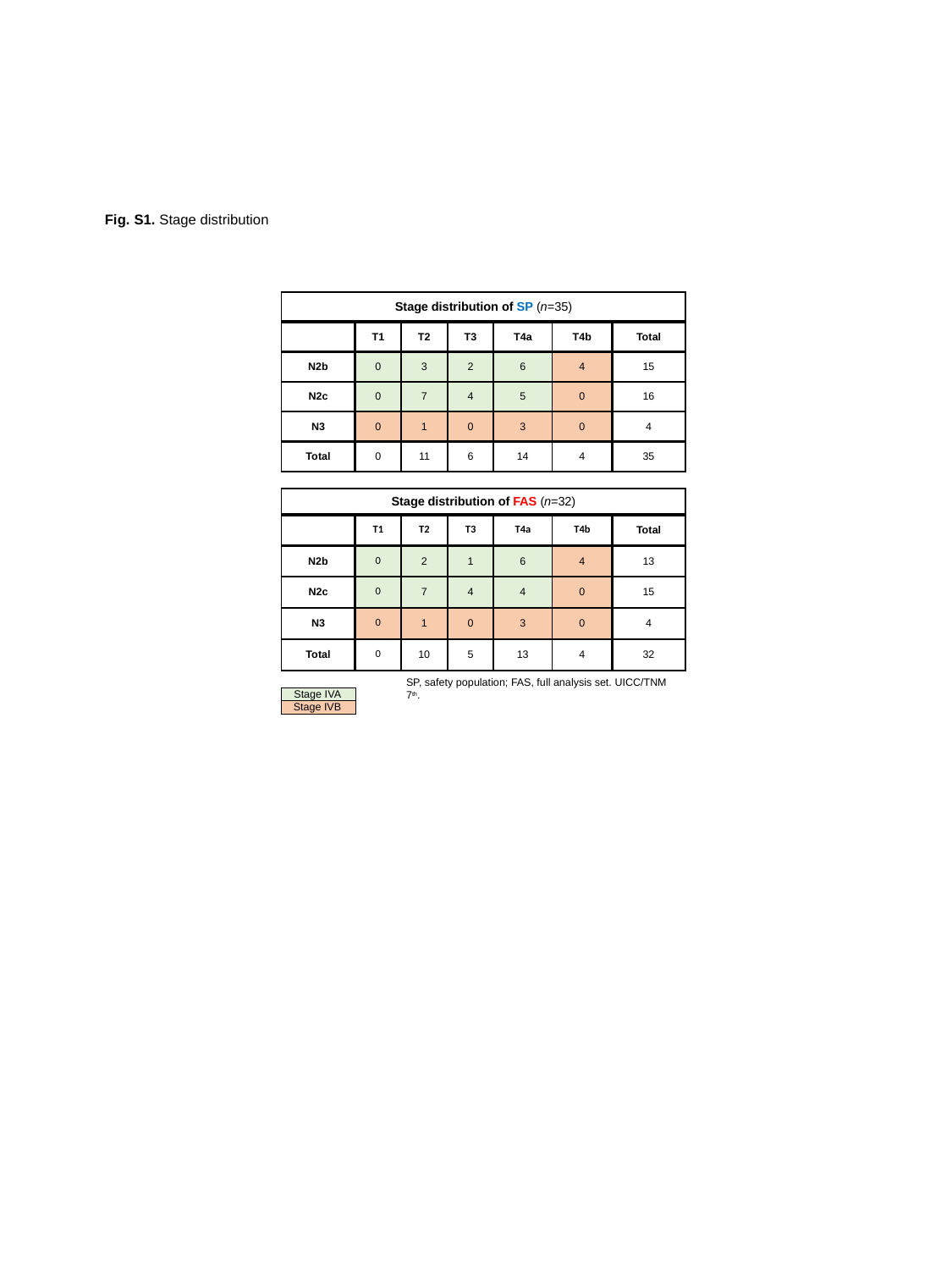

Fig. S1. Stage distribution
| Stage distribution of SP (n=35) | | | | | | |
| --- | --- | --- | --- | --- | --- | --- |
| | T1 | T2 | T3 | T4a | T4b | Total |
| N2b | 0 | 3 | 2 | 6 | 4 | 15 |
| N2c | 0 | 7 | 4 | 5 | 0 | 16 |
| N3 | 0 | 1 | 0 | 3 | 0 | 4 |
| Total | 0 | 11 | 6 | 14 | 4 | 35 |
| Stage distribution of FAS (n=32) | | | | | | |
| --- | --- | --- | --- | --- | --- | --- |
| | T1 | T2 | T3 | T4a | T4b | Total |
| N2b | 0 | 2 | 1 | 6 | 4 | 13 |
| N2c | 0 | 7 | 4 | 4 | 0 | 15 |
| N3 | 0 | 1 | 0 | 3 | 0 | 4 |
| Total | 0 | 10 | 5 | 13 | 4 | 32 |
SP, safety population; FAS, full analysis set. UICC/TNM 7th.
| Stage IVA |
| --- |
| Stage IVB |

## Slide 2
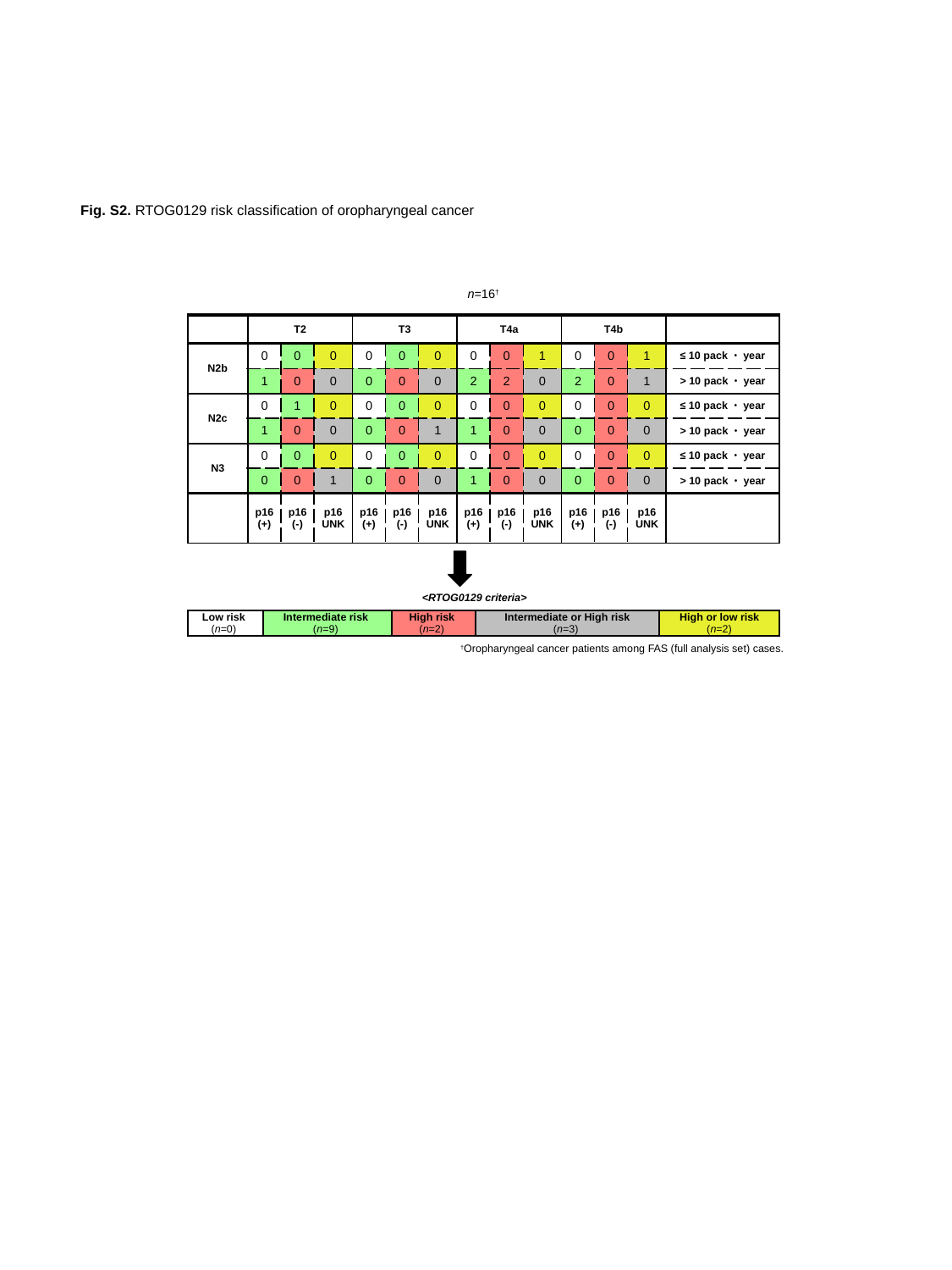

Fig. S2. RTOG0129 risk classification of oropharyngeal cancer
| n=16† | | | | | | | | | | | | | |
| --- | --- | --- | --- | --- | --- | --- | --- | --- | --- | --- | --- | --- | --- |
| | T2 | | | T3 | | | T4a | | | T4b | | | |
| N2b | 0 | 0 | 0 | 0 | 0 | 0 | 0 | 0 | 1 | 0 | 0 | 1 | ≤ 10 pack・year |
| | 1 | 0 | 0 | 0 | 0 | 0 | 2 | 2 | 0 | 2 | 0 | 1 | > 10 pack・year |
| N2c | 0 | 1 | 0 | 0 | 0 | 0 | 0 | 0 | 0 | 0 | 0 | 0 | ≤ 10 pack・year |
| | 1 | 0 | 0 | 0 | 0 | 1 | 1 | 0 | 0 | 0 | 0 | 0 | > 10 pack・year |
| N3 | 0 | 0 | 0 | 0 | 0 | 0 | 0 | 0 | 0 | 0 | 0 | 0 | ≤ 10 pack・year |
| | 0 | 0 | 1 | 0 | 0 | 0 | 1 | 0 | 0 | 0 | 0 | 0 | > 10 pack・year |
| | p16 (+) | p16 (-) | p16 UNK | p16 (+) | p16 (-) | p16 UNK | p16 (+) | p16 (-) | p16 UNK | p16 (+) | p16 (-) | p16 UNK | |
| <RTOG0129 criteria> |
| --- |
| Low risk (n=0) | Intermediate risk (n=9) | High risk (n=2) | Intermediate or High risk (n=3) | High or low risk (n=2) |
| --- | --- | --- | --- | --- |
†Oropharyngeal cancer patients among FAS (full analysis set) cases.

## Slide 3
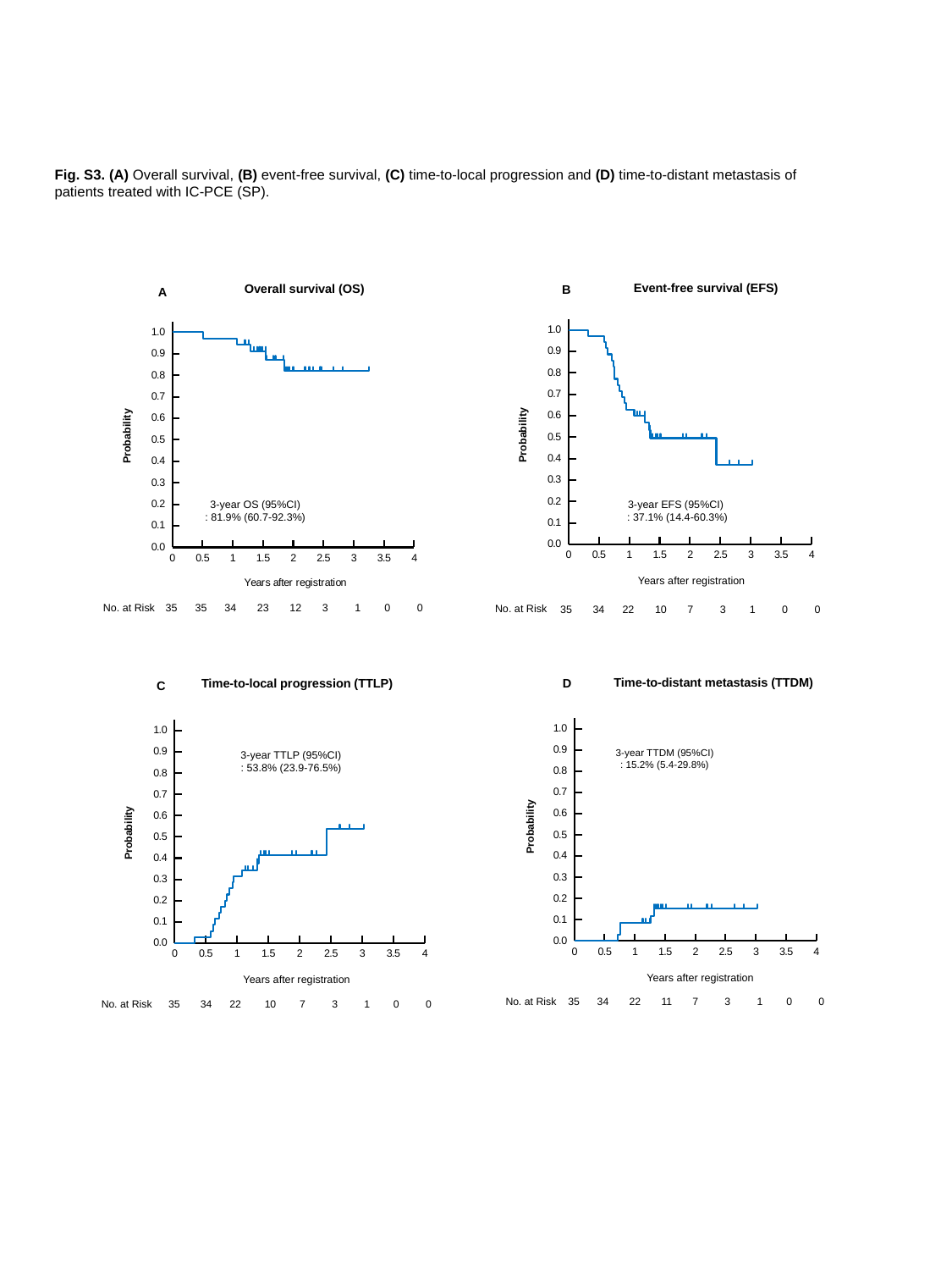

Fig. S3. (A) Overall survival, (B) event-free survival, (C) time-to-local progression and (D) time-to-distant metastasis of patients treated with IC-PCE (SP).
Event-free survival (EFS)
Overall survival (OS)
B
A
### Chart
| Category | |
|---|---|
### Chart
| Category | |
|---|---|Probability
Probability
3-year OS (95%CI)
: 81.9% (60.7-92.3%)
3-year EFS (95%CI)
 : 37.1% (14.4-60.3%)
Years after registration
No. at Risk
35 35 34 23 12 3 1 0 0
No. at Risk
35 34 22 10 7 3 1 0 0
Time-to-distant metastasis (TTDM)
Time-to-local progression (TTLP)
D
C
### Chart
| Category | |
|---|---|
### Chart
| Category | |
|---|---|3-year TTDM (95%CI)
: 15.2% (5.4-29.8%)
3-year TTLP (95%CI)
: 53.8% (23.9-76.5%)
Probability
Probability
Years after registration
Years after registration
No. at Risk
35 34 22 11 7 3 1 0 0
35 34 22 10 7 3 1 0 0
No. at Risk

## Slide 4
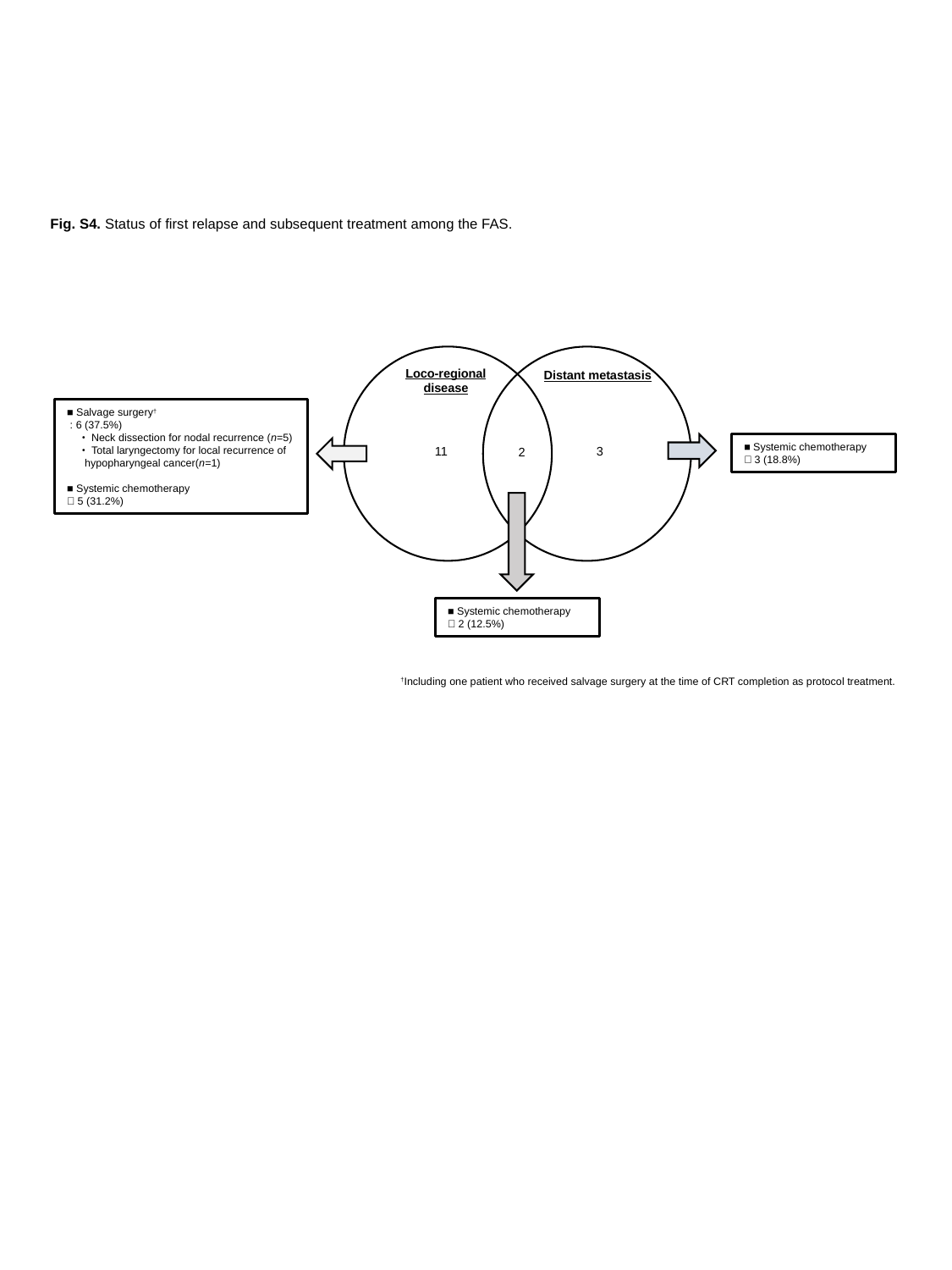

Fig. S4. Status of first relapse and subsequent treatment among the FAS.
Loco-regional
disease
Distant metastasis
■ Salvage surgery†
 : 6 (37.5%)
 ・Neck dissection for nodal recurrence (n=5)
 ・Total laryngectomy for local recurrence of
 hypopharyngeal cancer(n=1)
■ Systemic chemotherapy
：5 (31.2%)
■ Systemic chemotherapy
：3 (18.8%)
3
11
2
■ Systemic chemotherapy
：2 (12.5%)
†Including one patient who received salvage surgery at the time of CRT completion as protocol treatment.
