# Supplementary material for: A multicenter phase II trial of paclitaxel, carboplatin, and cetuximab followed by chemoradiotherapy in patients with unresectable locally advanced squamous cell carcinoma of the head and neck
Source: Cancer Med. 2020 Jan 13;9(5):1671–82. doi: 10.1002/cam4.2852 (PMC7050099; doi:10.1002/cam4.2852)
Supplement: Supplementary file 2 [file CAM4-9-1671-s002.docx]

**Table S1.** Compliance with induction chemotherapy

|  | **PTX** | | **CBDCA** | | | **Cmab** | |
| --- | --- | --- | --- | --- | --- | --- | --- |
|  | Current study | Haddad et al ^28^ | Current study | Haddad et al | | Current study | Haddad et al |
| **No. of administrations** | | | | | | | |
| Median (range) | 8 (3-8) | NA | 8 (3-8) | NA | | 8 (1-10) | NA |
| **Dose intensity^†^** | | | | | | | |
| Median (range) | 640 (240-640) | NA | 12 (4.5-12) | | NA | 2150 (400-2650) | NA |
| Mean | 604.9 | 724.29 | 11.3 | | 11.17 | 2095.7 | 1448.41 |
| **RDI**^‡^ | | | | | | | |
| Median (range) | 100 (37.5-100) | NA | 100 (37.5-100) | | NA | 100 (18.6-100) | NA |
| Mean | 90.5 | 89.95 | 90.8 | | 89.95 | 93.6 | 85.38 |

PTX, paclitaxel; CBDCA, carboplatin; Cmab, cetuximab, RDI: relative dose-intensity. NA, not applicable. ^†^Cumulative dose delivered during the induction phase. Units of measure are as follows: PTX, mg/m^2^; CBDCA, AUC (area under the curve), Cmab, mg/m^2^. ^‡^Ratio of actual dose intensity to planned dose intensity, accounting for treatment delays and dose reductions.

**Table S2.** Selected late toxicity

|  | *n*=29^†^ | | | | |
| --- | --- | --- | --- | --- | --- |
|  | **All Grades** | **Grade 1** | **Grade 2** | **Grade 3** | **Grade 4** |
| Osteonecrosis of jaw | 0 (0) | 0 (0) | 0 (0) | 0 (0) | 0 (0) |
| Laryngeal edema^‡^ | 14 (50) | 11 (39.3) | 2 (7.1) | 0 (0) | 1 (3.6) |
| Esophageal stenosis | 4 (13.8) | 2 (6.9) | 0 (0) | 2 (6.9) | 0 (0) |
| Dry mouth | 21(72.4) | 15 (51.7) | 6 (20.7) | 0 (0) | 0 (0) |
| Dysgeusia | 15 (51.7) | 14 (48.3) | 1 (3.4) | - | - |
| Peripheral sensory neuropathy | 4 (13.8) | 4 (13.8) | 0 (0) | 0 (0) | 0 (0) |
| Hypothyroidism^‡^ | 4 (14.3) | 3 (10.7) | 1 (3.6) | 0 (0) | 0 (0) |
| Dysphagia | 11 (37.9) | 7 (24.1) | 2 (6.9) | 2 (6.9) | 0 (0) |
| Hearing impairment | 3 (10.3) | 3 (10.3) | 0 (0) | 0 (0) | 0 (0) |
| **Total with ≥ Grade 3 toxicity** | 3 (10.3) | | | | |

Graded according to Common Toxicity Criteria for Adverse Events Version 4.0. ^†^29 FAS (full analysis set) patients received evaluation of late toxicity. ^‡^Data on laryngeal edema and hypothyroidism were available for 28 of 29 patients.
