# Supplementary material for: A multicenter phase II trial of paclitaxel, carboplatin, and cetuximab followed by chemoradiotherapy in patients with unresectable locally advanced squamous cell carcinoma of the head and neck
Source: Cancer Med. 2020 Jan 13;9(5):1671–82. doi: 10.1002/cam4.2852 (PMC7050099; doi:10.1002/cam4.2852)
Supplement: Supplementary file 3 [file CAM4-9-1671-s003.docx]

**Doc. S1.** Supplementary Methods (Patients and methods) **Patients
*Inclusion criteria***
(1) Histologically proven squamous cell carcinoma of the head and neck. 
(2) Primary lesion located at the larynx, oropharynx or hypopharynx.  
(3) Unresectable LASCCHN that fulfills at least one of the following conditions:
 (i) primary lesion or cervical lymph node metastasis invasion to carotid artery, cranial base, or cervical vertebrae
 (ii) cervical lymph node metastasis of N2b involving the lower neck (Level IV or supraclavicular lymph node), N2c or
 N3 (UICC⁄TNM, 7th edition)
 (iii) T4 primary lesion located at oropharynx.
(4) No cancer fistula due to primary lesion or cervical lymph node metastasis
(5) Measurable disease. 
(6) No evidence of distant metastasis. 
(7) ECOG Performance status (PS) of 0 or 1

(8) Age between 20 and 75 years. 
(9) Sufficient organ function. 
(10) HbsAg-negative. 
(11) No abnormal finding on electrocardiogram. 
(12) Women of child-bearing potential and men who are able to father a child agree to the use of adequate contraception. 
(13) Written informed consent to the study signed by the patient.

***Exclusion criteria***

(1) Primary lesion located at the nasopharynx, oral cavity, nasal cavity, parasinus or salivary gland. 
(2) Prior RT, CT or endocrine therapy for the current or any other malignancy. 
(3) Current other synchronous primary double cancers and metachronous double cancers unless free of disease for at least five years (excluding superficial cancer that will be cured by endoscopic mucosal resection). 
(4) Severe myelosuppression or infection. 
(5) Pulmonary fibrosis, acute lung injury or intestinal pneumonia. 
(7) Active gastrointestinal bleeding. 
(8) Clinically relevant comorbidity including heart failure, renal failure, liver failure, uncontrolled hypertension or uncontrolled diabetes mellitus. 
(9) History of severe hypersensitivity. 
(10) Known hypersensitivity against any components of the trial treatment, including excipients. 
(11) Pregnancy or breast feeding. 
(12) Current administration of disulfiram, cyanamide, carmofur or procarbazine hydrochloride. 
(13) Previous treatment with cetuximab or monoclonal antibody. 
(14) Other significant disease that in the investigator's opinion would exclude the subject from the trial.

**Treatment and assessment
*Radiotherapy***The GTV included the volumes of both the primary tumor and metastatic cervical lymph nodes with a short axis of 1 cm or larger. CTV1 included the GTV plus a 0.5–2 cm margin, and CTV2 included the prophylactic lymph node area. The PTVs for CTV1 and CTV2 (PTV1 and PTV2) were defined as the CTV plus 0.5–1-cm margins around CTV to compensate for set-up variation and internal organ motion. A total of 70 Gy was delivered to PTV1 (≥ 95% of the PTV1), while a prophylactic dose of 56 Gy (≥ 95% of the PTV2) was delivered to PTV2, using a simultaneous integrated boost (SIB) technique.

***Treatment modification and assessment***Doses of anti-tumor drug at the following cycle were modified according to the type of observed toxicities, as evaluated according to the Common Toxicity Criteria for Adverse Events version 4.0. The protocol treatment was terminated if there was a treatment delay of >14 days due to toxicity during IC. In the CRT phase, RT was omitted when severe toxicities were observed, and then restarted after the toxicities were resolved. Administration of CDDP was allowed within two weeks after the planned RT completion date. Baseline evaluation consisted of history, physical examination, upper gastrointestinal endoscopy, radiographic imaging, routine laboratory studies, and electrocardiogram. Safety assessments were repeated at least weekly during the protocol treatment. At the end of IC and 12 weeks after the completion of CRT, physical examination and radiographic imaging were performed to assess anti-tumor efficacy.

Good PR was characterized as a secondary change unique to post CRT, and defined as residual tissue with tumor shrinkage (scar material). It was further defined as all target lesions ≤10 mm in size and not enhanced on contrasted imaging examination. Good PR in this study was defined as all target lesions ≤10 mm in size and not enhanced on contrasted computed tomography scan. For patients with an objective response, including CR, good PR at the first evaluation after completion of CRT, additional treatment was not permitted unless recurrence was observed. When a patient had PR, SD or PD with consensus of persistent disease after completion of CRT, salvage surgery was considered as protocol treatment if it was judged to be clinically feasible. Efficacy and safety were evaluated at least every three months during the first year, at least every four months during the second year, and then every six months thereafter. Information on late toxicity was collected from subjects who were still alive and followed-up at the time of the data cut*-*off date. 

Criteria for discontinuation of protocol treatment

Protocol treatment is to be completed when the following criteria will be met;

1. Salvage surgery cannot be performed for PR, SD and PD after the completion of CRT.
2. Gross examination by the naked eye shows tumor tissue present at the resection 
   margin (macroscopic positive margin) at the time of salvage surgery as a protocol treatment.
3. Distant metastasis observed after the start of protocol treatment.
4. Grade 4 toxicity is observed except for hypocalcemia, hypocalcemia, hyperkalemia, hypokalemia, hypernatremia, hyponatremia, hypomagnesemia, hypophosphatemia after the start of protocol treatment.
5. Grade 3 encephalitis or grade 2 or higher leukoencephalopathy is observed after the start of protocol treatment.
6. Any one of “Criteria for initiation of chemotherapy” and “Criteria for initiation of cetuximab” are unmet within 14 days of postponement for reasons for which a relationship with adverse events cannot be ruled out during IC.
7. CRT cannot be started within four weeks after IC is completed.
8. Radiotherapy cannot be resumed beyond 21 days after postponement due to the reasons other than adverse events during CRT.
9. Death during protocol treatment.
10. Patient ineligibility come out after study entry.
11. Patient’s pregnancy is diagnosed after study entry.
12. Protocol treatment cannot be started within 14 days of study entry.
13. Consent withdrawal.
14. Continuation of protocol treatment is considered to be inappropriate by the physician-in-charge.

Criteria for initiation of 1^st^ cycle of PCE (CBDCA, PTX and Cmab) during IC

| **Characteristics** | **Criteria** |
| --- | --- |
| Neutrophil count | ≥ 1,500 /mm^3^ |
| Platelet count | ≥100,000/mm^3^ |
| Hemoglobin | ≥9.0 g/dL |
| Total bilirubin | ≤ 2.4 mg/dL |
| AST, ALT | ≤ 100 IU/L |
| Infection | No fever of ≥ 37.5°C suggestive of infection |
| Sensory neuropathy | Grade 2 or less |
| Ototoxicity | Grade 1 or less |
| Hand foot syndrome | Grade 1 or less |
| Hypomagnesemia | Grade 0 |
| Non-hematological toxicities | Grade 2 or less |
| Others | Investigator’s judge for postponement of chemotherapy |

Criteria for initiation of 2^nd^ or later cycle of chemotherapy (PTX, CBDCA) during IC

| **Characteristics** | **Criteria** |
| --- | --- |
| Neutrophil count | ≥ 1,000 /mm^3^ |
| Platelet count | ≥100,000/mm^3^ |
| Hemoglobin | ≥9.0 g/dL |
| Total bilirubin | ≤ 2.4 mg/dL |
| AST, ALT | ≤ 100 IU/L |
| Infection | No fever of ≥ 37.5°C suggestive of infection |
| Sensory neuropathy | Grade 2 or less |
| Non-hematological toxicities | Grade 2 or less |
| Others | Investigator’s judge for postponement of chemotherapy |

Criteria for initiation of 2^nd^ or later cycle of cetuximab during IC

| **Characteristics** | **Criteria** |
| --- | --- |
| Skin toxicity | Grade 2 or less |
| Hypomagnesemia | Grade 2 or less* |
| Others | Investigator’s judge for postponement of chemotherapy |

*Provided that, no grade 2 or worse prolongation of QTc is observed.

Dose modification during IC

- Criteria for dose reduction and discontinuation of chemotherapy (PTX, CBDCA)

When the following adverse events occur during previous cycle, dose of chemotherapy (PTX, CBDCA) will be reduced by 1 level for the subsequent cycle.

| **Criteria for dose reduction or discontinuation** | **Paclitaxel** | **Carboplatin** |
| --- | --- | --- |
| Grade 4 neutropenia lasting 8 days | Dose reduction | Dose reduction |
| Grade 3 febrile neutropenia | Dose reduction | Dose reduction |
| Grade 3 or worse thrombocytopenia | Dose reduction | Dose reduction |
| Grade 3 or worse hearing impairment | Not applicable | Discontinuation |
| Grade 2 peripheral sensory neuropathy | Dose reduction | Not applicable |
| Grade 3 peripheral sensory neuropathy | Discontinuation | Not applicable |
| Grade 3 or worse elevation of serum AST or ALT | Dose reduction | Not applicable |
| Grade 2 or worse elevation of serum bilirubin | Dose reduction | Not applicable |
| Other grade 3 or worse non-hematological toxicities | Dose reduction | Dose reduction |

・　Dose level of chemotherapy (PTX, CBDCA)

| **Lose level** | **Paclitaxel** | **Carboplatin** |
| --- | --- | --- |
| 0 | 80mg/m^2^ | AUC 1.5 |
| -1 | 64mg/m^2^ | AUC 1.2 |
| -2* | 48mg/m^2^ | AUC 1.0 |

*When further dose reduction is required after doses of the involved drugs are reduced by 2 levels from that at the initiation of protocol treatment, the administration of the drug is discontinued.

・　 Criteria for dose reduction and discontinuation of cetuximab
When the following adverse events occur during previous cycle, dose of chemotherapy will be reduced by 1 level for the subsequent cycle.

| **Criteria for dose reduction or discontinuation** | **Cetuximab** |
| --- | --- |
| Grade 3 or worse infusion reaction | Discontinuation |
| Grade 3 or worse cardiac toxicity | Discontinuation |
| Grade 3 or worse pneumonitis | Discontinuation |
| Grade 3 or worse allergic reaction | Discontinuation |
| Grade 3 or worse skin toxicities | Dose reduction |
| Grade 3 or worse hypomagnesemia^*^ | Dose reduction |
| Grade 2 hypomagnesemia with prolongation of QTc^*^ | Dose reduction |

^*^From the second time

・　Dose level of cetuximab

| **Lose level** | **Cetuximab** |
| --- | --- |
| 0 | 250 mg/m^2^ |
| -1 | 200 mg/m^2^ |
| -2 | 150 mg/m^2^ |

*When further dose reduction is required after doses of cetuximab reduced by 2 levels from that at the initiation of protocol treatment, the administration of cetuximab is discontinued.

Criteria for initiation of treatment during CRT

| **Characteristics** | **Criteria for**  **administration of CDDP^*^** | **Criteria for**  **omission of RT^**^** | **Criteria for**  **resuming of RT^*^** |
| --- | --- | --- | --- |
| Neutrophil count | ≥ 1,000 /mm^3^ | < 500 /mm^3^ | ≥ 500 /mm^3^ |
| Platelet count | ≥100,000/mm^3^ | < 25,000/mm^3^ | ≥ 25,000/mm^3^ |
| Creatinine clearance | ≥ 40 ml/min | - | - |
| Total bilirubin | ≤ 2.5 mg/dL | - | - |
| AST, ALT | ≤ 100 IU/L | - | - |
| Infection | Grade 0 | Fever of ≥ 37.5°C suggestive of infection | No fever of ≥ 37.5°C suggestive of infection |
| Febrile neutropenia | Grade 0 | Grade 3 or worse | Grade 0 |
| Radiation dermatitis | Grade 3 or less | Grade 4 or worse | Grade 3 or less |
| Mucositis | Grade 3 or less | Grade 4 or worse | Grade 3 or less |
| Others | Investigator’s judge for postponement of treatment | | |

*There is a need to meet all. **Any one of them.

Dose modification of CDDP during CRT

- Criteria for dose reduction and discontinuation of CDDP

When the following adverse events occur during previous cycle, dose of chemotherapy will be reduced by 1 level for the subsequent cycle.

| **Criteria for dose reduction or discontinuation** | **Cisplatin** |
| --- | --- |
| Grade 4 leukopenia, neutropenia, thrombocytopenia | Dose reduction |
| Grade 3 febrile neutropenia | Dose reduction |
| Grade 2 sensory neuropathy | Dose reduction |
| Decreased in creatinine clearance (<40 ml/min ) | Discontinuation |
| Decreased in creatinine clearance (40 – 49 ml/min ) | Dose reduction (level -2) |
| Decreased in creatinine clearance (50 – 59 ml/min ) | Dose reduction (level -1) |
| Grade 3 or worse hearing impairment | Discontinuation |
| Grade 2 hearing impairment | Dose reduction |
| Other non-hematological toxicities | Discuss with the study committee if necessary. |

・ Dose level of CDDP

| **Lose level** | **Cisplatin** |
| --- | --- |
| 0 | 20 mg/m^2^ |
| -1 | 16 mg/m^2^ |
| -2 | 12 mg/m^2^ |

*When further dose reduction is required after doses of CDDP reduced by 2 levels from that at the initiation of protocol treatment, the administration of CDDP is discontinued.

**Study design**
The secondary endpoints of this study were objective response rate (the proportion of CR and PR in IC phase, and CR, good PR and PR in CRT phase), clinical complete remission rate (the proportion of CR and good PR), time-to-local progression (TTLP), time-to-distant metastasis (TTDM), event-free survival(EFS), overall survival (OS) and adverse events. TTLP was defined as the time from enrolment to local disease progression. TTDM was defined as the time from enrolment to distant disease progression. EFS was defined as the time from enrolment to any disease progression or death from any cause. OS was defined as days from enrolment to death from any cause. All the above time-to-event endpoints were censored at the last follow-up date. For local progression, distant disease progression and deaths were treated as competing risks.

**Statistics**We retrospectively reviewed individual data of the following 75 patients who had undertaken CDDP-based CRT (CDDP at a dose of 20 mg⁄m^2^⁄day on days 1-4, repeated three times at 3-week intervals, which is identical to that of current study) without any induction chemotherapy at National Cancer Center Hospital East to determine the threshold and expected values of %CRT completion.
Patient characteristics of reference cohort (N=75)

|  | **No. of Patients (%)**  January 2008 - February 2013 |
| --- | --- |
| **RT planning**   IMRT  3D-RT | 67 (89.7%)  8 (10.7%) |
| **RT regimen**  2Gy/Fr | 75 (100%) |
| **Primary site**  Hypopharynx  Oropharynx  Oral cavity  Larynx | 28 (37.3%)  20 (26.7%)  19 (25.3%)  8 (10.7%) |
| **PEG placement**   Yes   No | 71 (94.7%) 4^†^ (5.3%) |

RT, radiotherapy; IMRT, intensity-modulated radiotherapy; 3D-RT, 3 dimensional-RT; PEG, percutaneous endoscopic gastrostomy. ^†^Two of these patients had enterosromy instead of PEG.

An interim analysis was planned when %CRT completion of the first enrolled 23 patients became available, but the analysis was shelved because an enrollment was much faster than expected. While safety population (SP) was defined as patients who received at least one dose of any study product. For %CRT completion, in addition to Bayesian PP, we report (1) crude proportion and its 95% confidence interval (CI) with the use of Clopper and Pearson method and (2) average of posterior distribution together with 95% Bayesian probability interval. For OS and EFS, the survival curves were estimated using the Kaplan-Meier method. For TTLP and TTDM, the cumulative incidence curves were estimated using a standard survival analysis method for competing risks. We did post-hoc subgroup analyses for OS, focusing on primary site (oropharyngeal primary vs. others), p16 positivity of oropharyngeal cancer and CR vs. good PR after completion of CRT. Because of the limited number of events, we just compared survival curves and employed the log-rank test for reference. All statistical analyses were carried out using SAS Release 9.4 (SAS Institute, Cary, NC, USA).
